# The Water Question

**Published:** 1895-09

**Authors:** R. M. Swearingen


### EDITORIAL DEPARTMENT.

F. E. DANIEL, M. D., Editor.
S. E. HUDSON, M. D., Managing Editor.
A. J. SMITH. M. D., Galveston, Associate Editor.

EDITORIAL STAFF:

PROF. J. E. THOMPSON, M. D., Texas Medical College, Galveston; Surgery.
PROF. WM. KEILLER, M. D., Texas Medical College, Galveston; Obstetrics and Gynecology.
PROF. DAVID CERNA, M. D., Texas Medical College, Galveston; Therapeutics.
PROF. A. J. SMITH, M. D., Texas Medical College, Galveston; Medicine
DR. R. H. L. BIBB, Saltillo, Mexico; Foreign Correspondent.

Official organ of the West Texas Medical Association, the Houston District Medical Association, the Austin District Medical Society, the Galveston County Medical Society, and several others

### THE WATER QUESTION.

The citizens of Austin are laboring with a sanitary problem of no small proportions. Having constructed a granite dam across the raging Colorado, at a cost of about a million and a half dollars, and having, as a preliminary to said construction, built a railroad to the spot at a cost of, say, a hundred thousand dollars, they succeeded in creating a first class pleasure resort, and facilities for pleasure excursions, which threaten now to defeat, in a measure, a part of the prime object for which the gigantic work was undertaken; i. e., to supply Austin with water and light, and incidentally to furnish water power for machinery, should factories be established here. The light system and the water system are complete and in operation, but lo, and behold, the cry is raised, and with good cause, the water in the lake, whence the supply is to be drawn for all purposes, has become, and will continue to be, contaminated by the uses to which the lake is put, and by the proximity of the military encampment, which is located on the water shed leading to the lake. The big steamboat, running moonlight and other excursions, carrying hundreds of people each trip, and making many trips, and the smaller steamboats and other crafts; the camping and picnic parties along the borders, are all sources of contamination, and the Austin people are scared up about drinking the water; although there has occurred no typhoid fever or other disease here to justify such fears, there is ground for apprehensions that such will result in future.

It becomes necessary, then, to both clarify the water, as, especially after a rise, it is muddy, and purify it; and this is the question now agitating the Austin public: how can it best be done? The citizens cheerfully submitted to an additional burden, in the way of another bond issue, to raise money enough to construct a reservoir; that seems to be the main idea, and the only thing that suggests itself as a solution to the problem. It is proposed to take advantage of the natural formation of the ground above the lake, and dam up one side of a ravine, thus creating a tank some distance higher than the lake level, and with a capacity sufficient to keep stored water enough to meet the demands; and this tank, it is estimated, will cost anywhere from $45,000 to $100,000.

Again, the objection is raised: if the water, at its source, be impure, how will the mere shifting of its position, and storing it in a tank, benefit it? There it will be entirely stagnant, whereas in the lake the current was doing something towards purification, and it will be subjectd to the action of influences calculated to still fnrther injure it as drinking water—the action of the sun on a limited surface, the growth of vegetable and parasitic matter, etc., incidental to stagnation—to say nothing of impurities washed in from the adjacent hills, unless great precautions are taken.

It is very evident, therefore, that something else must be done in addition to, or in lieu of building a reservoir.

State Health Officer Swearingen who, in addition to being an advanced sanitarian and an authority, is a public-spirited citizen, voluntarily offered suggestions to the authorities, in a letter published in the *Statesman*, which suggestions, as they deal with the question in the abstract, have more than a local application, and we herewith reproduce the letter for the benefit of our readers, and of any other city which may be wrestling with the water supply question. It is a question of vital importance, and too much attention can not be given to its stady:

*Editor Statesman:*—The trend of human effort in the line of safeguards and protective sanitary measures, keeps step with the highest civilization, and marks the progress of a people's intelligence.

The health authorities of great cities are forever wrestling with the question of pure water, and inspectors guard with never ceasing vigilance every avenue of pollution. The laws of New York State give the water commissioners of the city authority to cement vaults and close sewers over the entire water shed of the Croton river, an area of 340 miles. With all these precautions, with abundant means, and the most skillful experts, New York has failed, as have all other large cities, to secure perfectly pure drinking water.

Scientific investigations, however, have demonstrated one great comforting fact: that these sanitary measures do give good results, and that sickness increases or diminishes as these laws are neglected or enforced.

Reliable vital statistics had established this sanitary truth, and it had been universally accepted, long years before the microscope presented its incontrovertible proof. The discoveries of Dr. Koch, in 1883, of the comma bacillus, gave a new impulse to sanitary science, and the knowledge that cholera and typhoid fevers were disseminated and propagated by contaminated waters, made the uneducated halt and reflect.

When the supply streams have been unavoidably impure, ingenuity has been taxed to correct the evil and lessen the danger. The stone filter jar was first tried in private families, and gave so much satisfaction that factories and hotels enlarged them to meet their requirements, and so the idea grew until towns and cities had them constructed capable of passing one, two, three and four million gallons of water through them per day.

Sand vats were first introduced by James Simpson, in 1839, near London. The Thames river had furnished the supply, and for several years before filtration by the vats, and several years thereafter, Professor Frankland had made a series of carefully managed experiments. The average results of these experiments for 1886, 1887 and 1888, shows that the sand had caught 97 per cent of the bacteria, and reduced the death rate of typhoid fever, which had before those years been very heavy, to 1.6 per 10,000 inhabitants.

These figures, and many others like them, make a strong argument for the sand vat system.

In a turbid stream, like the Colorado, it would be almost impossible to keep these artificial sand beds in proper condition. During a heavy freshet, particularly a red rise, there would be an ounce of mud to every gallon of water passed through the sand vat, and estimating a million of gallons per day, would leave us a veneering of 62,000 pounds of red mud on top of the sand that would exclude all chance of filtration until removed.

These rises occur too often and last too long for the sand vats to be classed among the possibilities.

The impracticability, however, of having these artificial sand filters, forced ingenuity into the easier, cheaper, and probably better method, the one known as the "driven well" system, now operated at Buffalo, New York, and, on a much smaller scale, by the old water company of this city. It consists in sinking iron tubes, or pipes, with many small holes in the lower end, deep into a sand bank—in fact, deep enough to tap a subterranean current that percolates through the white sand (in our case probably an old bed of the river) thirty or forty feet from the surface; through these tubes, a constant stream of clear water is brought, that is apparently free from organic matter. It is filtration through natural sand banks instead of artificial, and it has this, to me, unexplainable but weighty advantage: the mud held in solution by freshets never seems to close the avenues to these subteranean sand beds, nor do they lessen the free percolations of water through them. Whether the sand beds between the dam and the city can be so utilized, can only be determined by the practical experiments of capable men.

I have no facts to predict an estimate of the cost of such a plant, but it is reasonable to assume that it would be less expensive than any other that would promise as good results. The reservoir is the question of the hour, and is worthy of thoughtful consideration. Our granite dam is a marvel of engineering skill, and will tell to distant ages the pluck of a noble people bravely trying to better their condition. Lake McDonald mirrors the greenest banks and loveliest scenery of the earth, and furnishes a water supply sufficient for a population an hundred times greater than our city. But the beauty and ever-varying charms of this inland sea is a menace to public health. The carnivals and regattas, military encampments and moonlight excursions are drawing cards, and make the Capital City the most attractive one in the State. All the so-called progressive citizens favor these delightful gatherings, and the old fogy sanitarian who refuses to encourage them, is regarded by many as disloyal and selfish. In the face of such an indictment, I for one, enter an earnest protest. These pleasure seekers are unrestrained by any kind of police regulations, and they treat all sanitary laws that protect humanity from dangerous diseases with reckless indifference. Our lake, instead of being protected from every form of pollution by her lawful guardians, is made a splendid cess pool, where all kinds of micro-organisms can be planted and cultivated, and then drank and drank by happy, progressive thousands. Other cities close up and cement cess pools, while we float them on the bosom of the lake and build them over its water shed, knowing that all excrementitious matters will be forced by the currents straight toward the mains leading to the city.

Sedimentation is a prominent factor in the process of purification, and of course accomplishes some good.

It is the natural settling of inorganic matters and all particles that can be overcome by gravitation. As these myriads of atoms sink to the bottom they carry with them great bodies of organic matters, living and dead, to their final resting places.

Where a rapidly flowing current reaches a slower current, the tendency of all matters held in solution is toward the bottom. The bars and banks forming at the upper end of the lake are made in obedience to this physical law. It will in the course of time close up the greater portion of the basin. When it does accomplish that inevitable task, no great injury will result, for the power and volume of water will remain. The process of sedimentation that unceasingly works for the purification of the water, from the source to the mouth of streams, can be more effiectually accomplished when the water is stored in a reservoir and becomes almost motionless.

When these natural helpmates are supplemented by double cylinder filters—one above the other—with self-adjusting apparatus for cleaning, for all practical requirements we might claim an ideal water supply.

But will this water, so stored, keep pure, you ask, under midsummer suns? I answer, if malodorous water is the result of standing still, it must be caused by the decomposition of organic matters, and if by sedimentation and filtration we eliminate these organisms, it logically follows that we will not have offensive water, when it is stored in a reservoir, carefully constructed and possessing all the modern improvements for filtering and cleaning.

Assuming that the positions herein taken are true, the question resolves itself into one of finance. Money can give us a healthful, splendid reservoir, but it will, in my judgment, require a much larger sum than we have at our disposal to build one at the Brackenridge site, with all the new equipments and environments necessary to perfect it.

The city council has exclusive control of the business, and should they decide adversely to the reservoir, on account of not having sufficient funds, the driven well system with supply tubes and metal standpipes will doubtless receive consideration. In a newspaper article we can not go into details and elaborate systems; but the subject is worthy of profound study.

These suggestions are offered in no advisory spirit, but with an earnest desire to invite further discussion and to invoke all the lights of modern science around the complex vital problem.

R. M. SWEARINGEN.

